# Imputing forest carbon stock estimates from inventory plots to a nationally continuous coverage

**DOI:** 10.1186/1750-0680-8-1

**Published:** 2013-01-11

**Authors:** Barry Tyler Wilson, Christopher W Woodall, Douglas M Griffith

**Affiliations:** 1 USDA Forest Service, Northern Research Station, Forest Inventory and Analysis, St. Paul, MN, 55108, USA

**Keywords:** Forest, Carbon density, Imputation, United States, Forest inventory, Raster maps

## Abstract

The U.S. has been providing national-scale estimates of forest carbon (C) stocks and stock change to meet United Nations Framework Convention on Climate Change (UNFCCC) reporting requirements for years. Although these currently are provided as national estimates by pool and year to meet greenhouse gas monitoring requirements, there is growing need to disaggregate these estimates to finer scales to enable strategic forest management and monitoring activities focused on various ecosystem services such as C storage enhancement. Through application of a nearest-neighbor imputation approach, spatially extant estimates of forest C density were developed for the conterminous U.S. using the U.S.’s annual forest inventory. Results suggest that an existing forest inventory plot imputation approach can be readily modified to provide raster maps of C density across a range of pools (e.g., live tree to soil organic carbon) and spatial scales (e.g., sub-county to biome). Comparisons among imputed maps indicate strong regional differences across C pools. The C density of pools closely related to detrital input (e.g., dead wood) is often highest in forests suffering from recent mortality events such as those in the northern Rocky Mountains (e.g., beetle infestations). In contrast, live tree carbon density is often highest on the highest quality forest sites such as those found in the Pacific Northwest. Validation results suggest strong agreement between the estimates produced from the forest inventory plots and those from the imputed maps, particularly when the C pool is closely associated with the imputation model (e.g., aboveground live biomass and live tree basal area), with weaker agreement for detrital pools (e.g., standing dead trees). Forest inventory imputed plot maps provide an efficient and flexible approach to monitoring diverse C pools at national (e.g., UNFCCC) and regional scales (e.g., Reducing Emissions from Deforestation and Forest Degradation projects) while allowing timely incorporation of empirical data (e.g., annual forest inventory).

## Background

Forest ecosystems represent the largest terrestrial carbon (C) sink on earth [1,2], such that the United Nations Framework Convention on Climate Change [3] has recognized their management as an effective strategy for offsetting greenhouse gas (GHG) emissions [4,5]. As part of the Convention, the U.S. has been submitting national reports, the National Greenhouse Gas Inventory (NGHGI), detailing emissions and removals of GHGs [3] on an annual basis for many years [6]. In addition to international reporting requirements, GHG budgets are being developed at sub-national scales including states (e.g., California) and ownerships (e.g, National Forest System climate change scorecard). Forest C stocks in the U.S. are estimated using data from the national forest inventory conducted by the USDA Forest Service, Forest Inventory and Analysis (FIA) program [7]. Broad forest ecosystem components (e.g., aboveground live biomass) have been delineated to generalize C stocks to meet international reporting agreements pursuant to refining understanding of global carbon cycling [2,3]. Carbon estimates for the ecosystem components of forest floor (inclusive of litter, fine woody debris, and humic soil horizons), down dead wood, belowground (BG) biomass, and soil organic matter are calculated by FIA using models based on geographic area, forest type, and, in some cases, stand age [6,8]. Estimates of aboveground (AG) standing live and dead tree C stocks are based on biomass estimates obtained from inventory tree data [6,9]. Although forest C stock estimates, such as those from FIA, are readily available at national and regional scales [6,7], there is increasing interest in disaggregating these large-scale numerical estimates into maps of continuous estimates to enable strategic forest management and monitoring activities geared toward offsetting GHG emissions [10] and advancing C dynamics research.

Secondary to the need for spatially continuous forest C maps, numerous constituents (e.g., managers, policy makers, and scientists, forest analysts) require an efficient methodology for incorporating annual monitoring information into C maps. Sophisticated approaches to mapping forest C stocks may provide robust estimates of stocks [11], but lack the flexibility to rapidly incorporate annual monitoring information. As numerous forest C pools may change on annual time steps, especially in response to stochastic disturbance events, temporal accuracy of C maps may often be of equal importance as the need for spatial accuracy. Woodall et al. [12] found that actual standing dead tree C stocks were often significantly different than those modeled for the same inventory plots. Despite the measurement/model error associated with annual forest inventory programs, the temporally dynamic nature of forest ecosystems (e.g., wildfires and wind events) necessitates the incorporation of annual data into map products employed by scientists and stakeholders alike.

Wilson et al. [13] developed a methodology (hereafter referred to as Phenological Gradient Nearest Neighbor, or PGNN, for convenience) for producing maps of tree species occurrence and relative abundance over large areas by utilizing information collected on FIA field plots in conjunction with 250 m pixel resolution raster data in a *k*-nearest neighbor (*k*NN) imputation framework. The PGNN approach builds upon the Gradient Nearest Neighbor (GNN) work of Ohmann and Gregory [14], who integrated nearest-neighbor imputation of FIA plots with ecological ordination via canonical correspondence analysis (CCA). PGNN is best described as a hybrid of the *k*NN and GNN approaches, since it also makes use of CCA but utilizes *k* nearest neighbors during imputation rather than only a single neighbor. Another distinguishing characteristic is that it utilizes vegetation phenology information derived from multi-temporal satellite imagery, as well as climate, topographic, and ecoregion data compiled at a 250 m pixel resolution. One of the most attractive features of this approach is the efficiency with which a plot identification map can be produced at the national scale. In other words, every pixel is assigned a forest inventory plot label as well as the attributes of the labeled plot’s nearest neighbors, as defined by the CCA model. In the case of forest C accounting, every pixel could be assigned C stock estimates in a rapid fashion on an annual time-step.

Given the need for C maps at the national scale and the possible application of PGNN, the goal of this study was to apply PGNN for imputing national forest inventory plots to a spatially continuous raster grid in order to produce mapped estimates of the conterminous U.S.’s forest C density with these specific objectives: 1) to produce and interpret maps of forest carbon density by individual pools and combinations thereof (total forest ecosystem C density, live tree AG, live tree BG, live understory AG and BG, standing dead tree AG, downed dead wood, forest floor, soil organic carbon, and the pool that has the highest proportion of total forest ecosystem C density); 2) to conduct validation of the C mapping approach by comparing map-based and field plot-based estimates using a variety of metrics; and 3) to suggest future research directions and applications.

## Results

**Figure 1:**
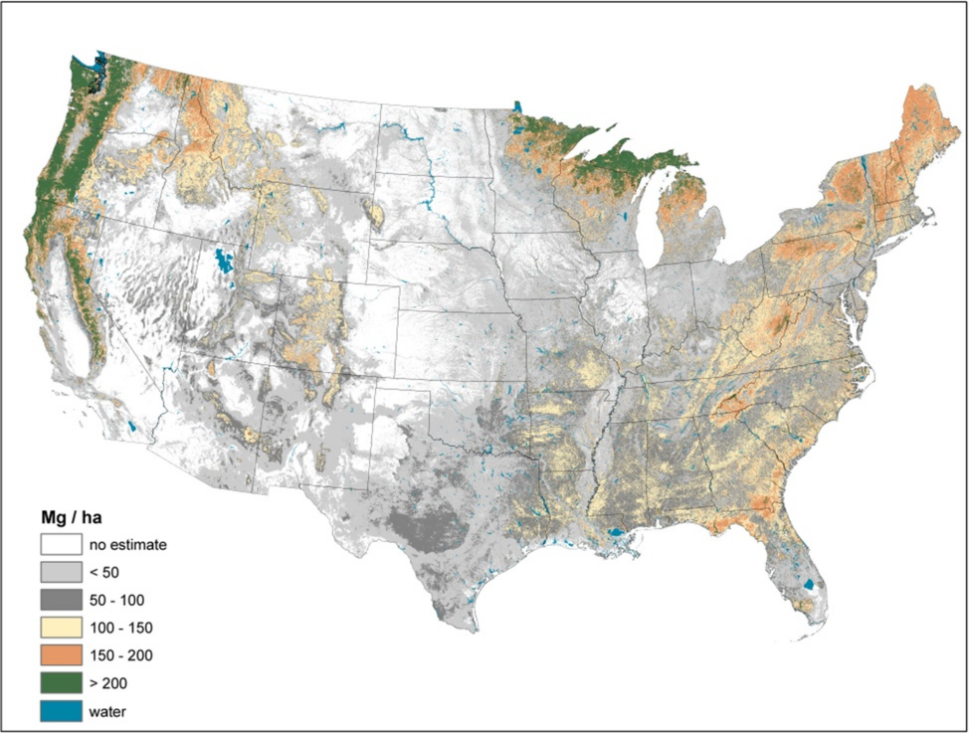
**Total forest ecosystem carbon density imputed from forest inventory plots, conterminous U.S., 2000-2009.** Includes above- and belowground live trees, downed dead wood, forest floor, soil organic carbon, standing dead trees, understory above- and belowground pools.

The imputed raster maps of total forest ecosystem C stocks (i.e., sum of all pools) suggest a rather disparate distribution of large total C stocks across the U.S. (Figure 1). While most forested areas of the U.S. have moderate C stock density (< 100 Mg/ha) (e.g., lower elevations of the Rocky Mountains, Central, and Plains states), there are other areas that have C densities in excess of 200 Mg/ha such as the Pacific Northwest and the upper Great Lakes. As total C stocks are comprised of diverse forest ecosystem components, examining the distribution of C stock density by component can refine understanding of C density dynamics at a national scale.

**Figure 2:**
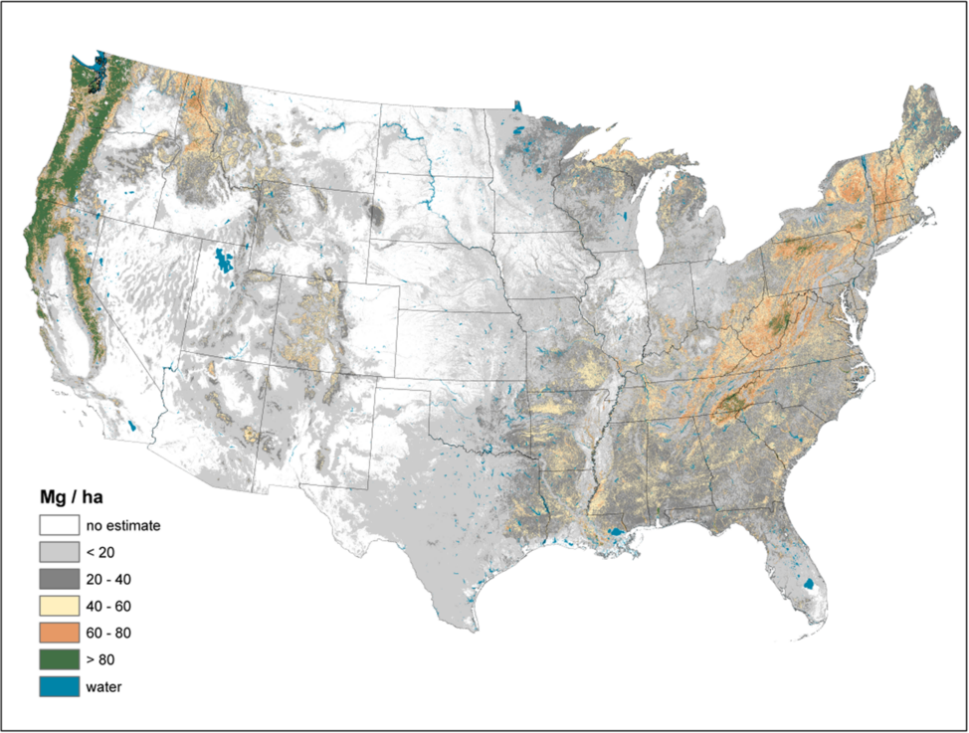
Live tree aboveground carbon density imputed from forest inventory plots, conterminous U.S., 2000-2009.

**Figure 3:**
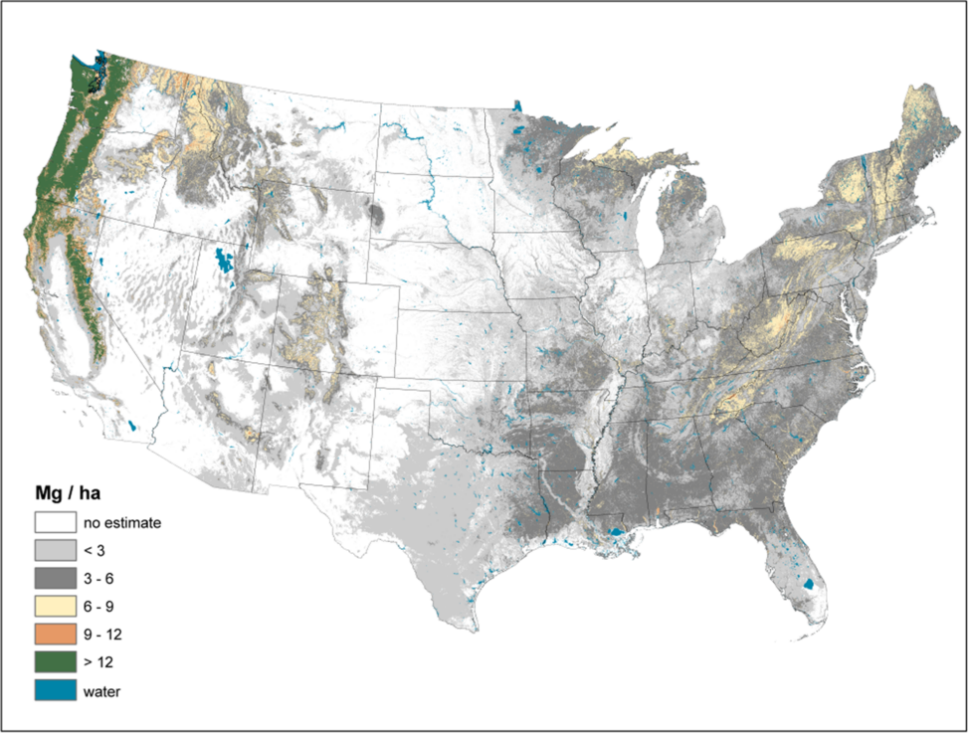
Live tree belowground carbon density imputed from forest inventory plots, conterminous U.S., 2000-2009.

**Figure 4:**
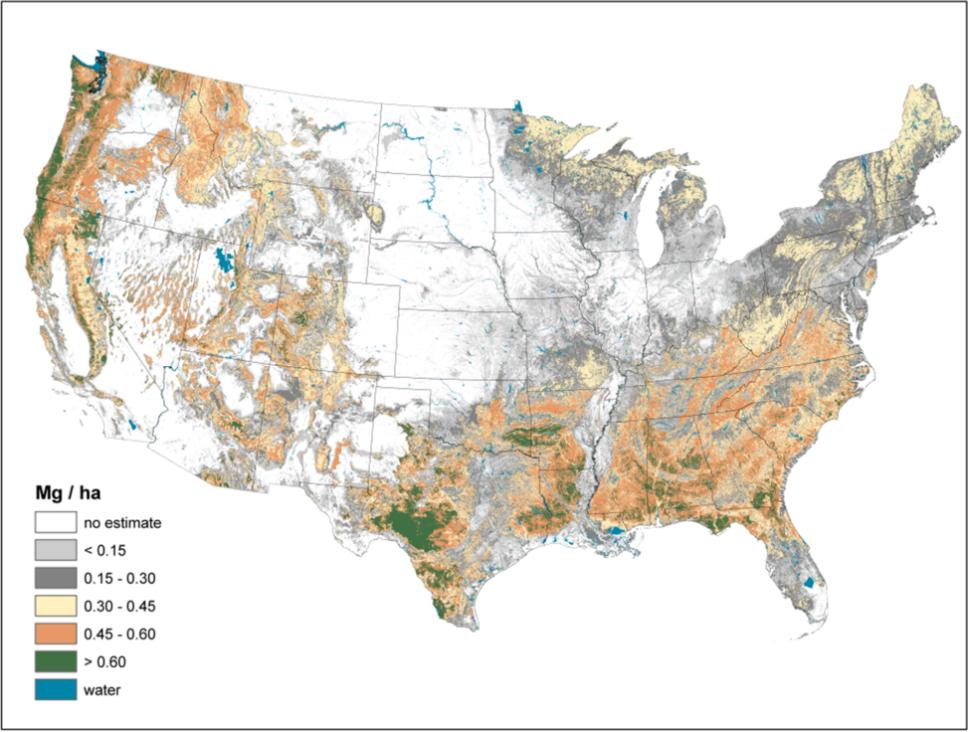
Live understory above and belowground carbon density imputed from forest inventory plots, conterminous U.S., 2000-2009.

**Figure 5:**
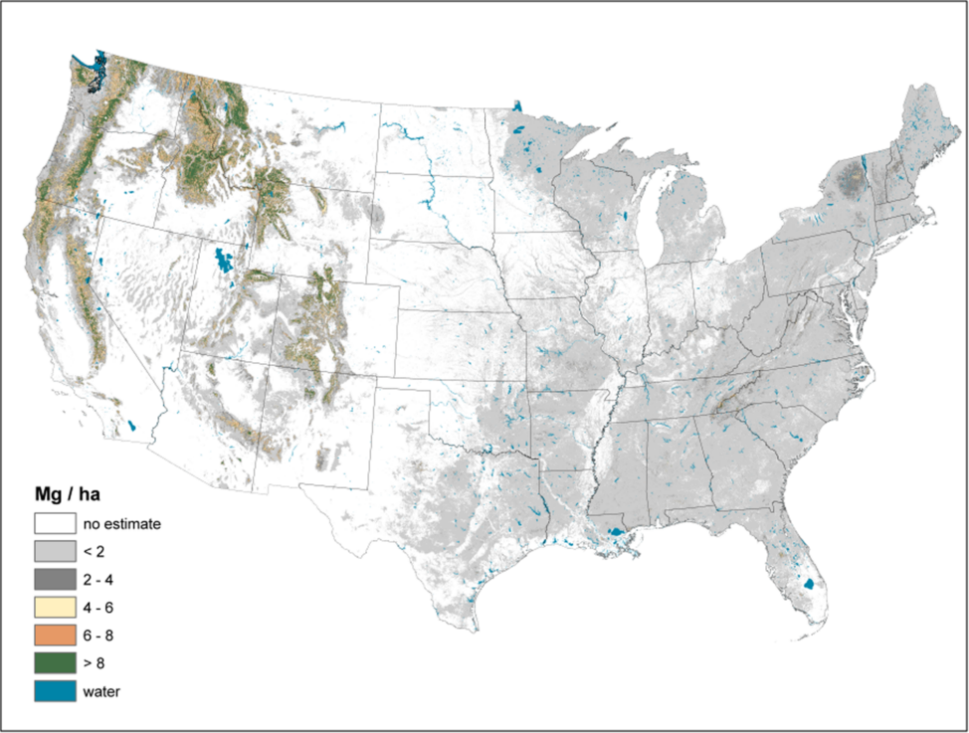
Standing dead tree above ground carbon density imputed from forest inventory plots, conterminous U.S., 2000-2009.

**Figure 6:**
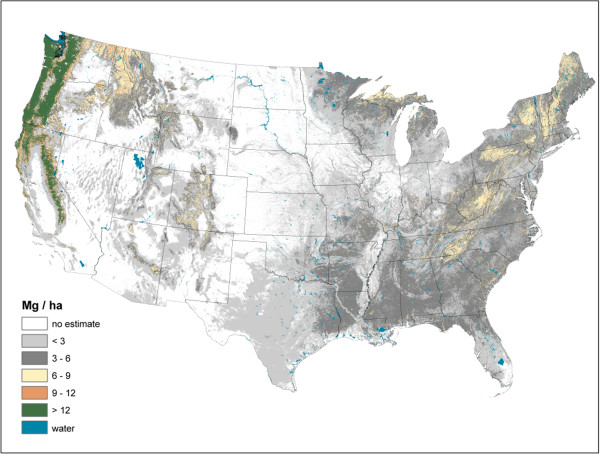
Downed dead wood carbon density imputed from forest inventory plots, conterminous U.S., 2000-2009.

**Figure 7:**
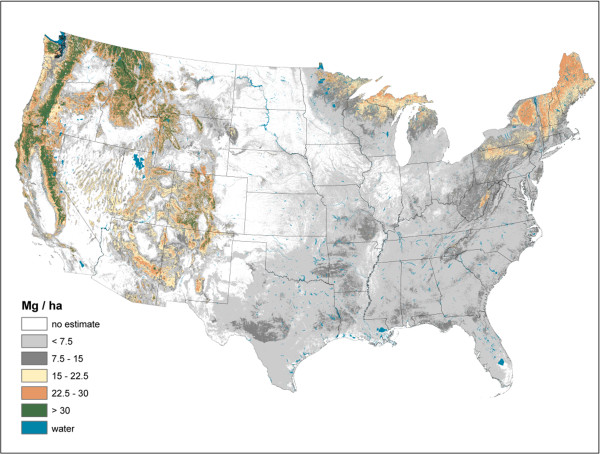
Forest floor carbon density imputed from forest inventory plots, conterminous U.S., 2000-2009.

**Figure 8:**
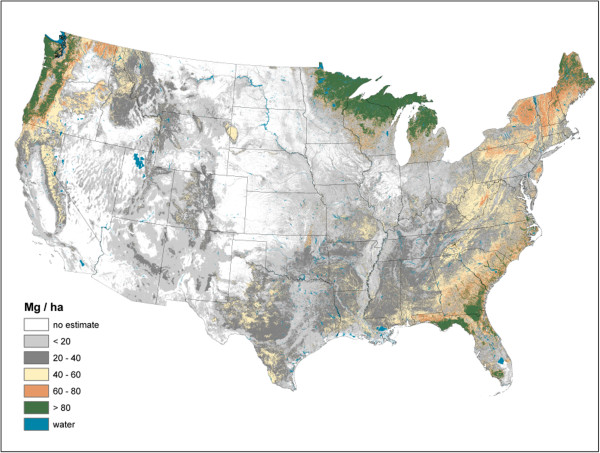
Soil organic carbon density imputed from forest inventory plots, conterminous U.S., 2000-2009.

**Figure 9:**
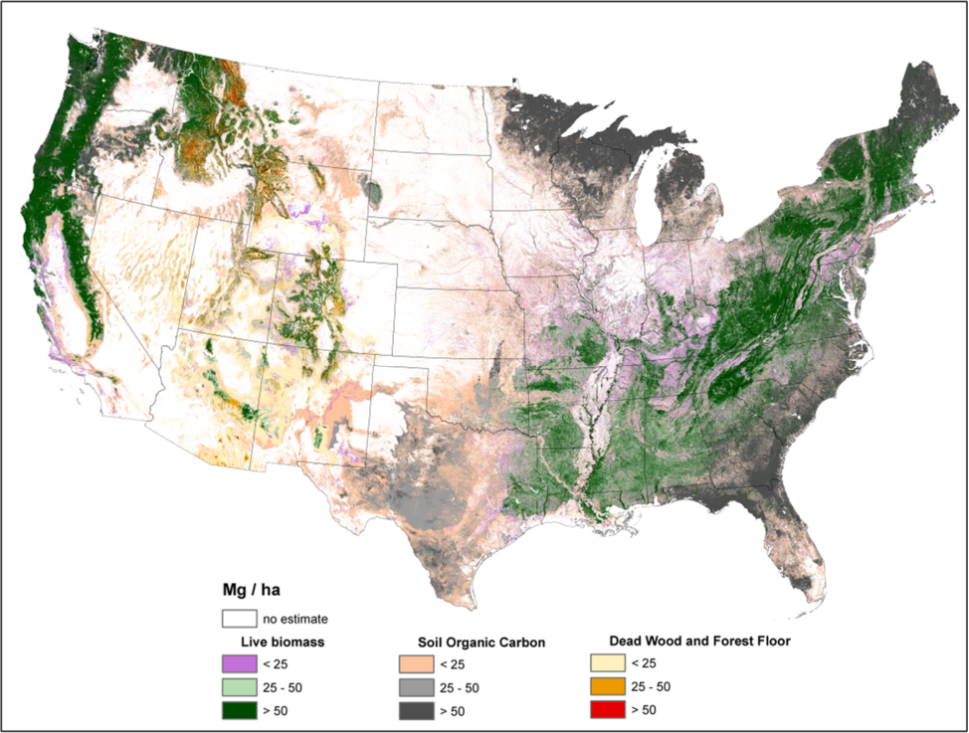
**Major forest carbon pools with the plurality of total forest carbon stock for each pixel imputed from forest inventory plots, conterminous U.S., 2000-2009.** Major pools are: 1) living biomass (aboveground, belowground, and understory), 2) dead wood and forest floor (including standing dead, down dead, and forest floor), and 3) soil organic carbon.

Live AG and BG (Figures 2 and 3) C stock density is highest in the Pacific Northwest, northwest California, northern Rockies, and Appalachian Mountains. The highest live AG C stock density often exceeds 80 Mg/ha. As BG C stocks are modeled as a function of AG C stocks, their spatial distributions are closely aligned. Live understory AG and BG C density follow spatial patterns in allocation similar to live tree distributions, albeit at a much lower density (< 1 Mg/ha) (Figure 4). Standing dead tree C stock densities are highest (> 8 Mg/ha) in the Olympic Mountains, Cascade Range, and North and Central Rocky Mountains (Figure 5). In comparison to the western U.S., eastern standing dead tree C stock density is minimal with only the Adirondacks and isolated areas of the Appalachian Mountains having a stock density exceeding 2 Mg/ha. The highest downed dead C stock densities (> 12 Mg/ha) are almost exclusively found in the Pacific Northwest and West Coast/Sierra Nevada (Figure 6). The detrital components of forest floor and SOC have spatial distributions fundamentally different from woody biomass C stock distributions (Figures 7 and 8). The highest C stock densities for forest floor (> 15 Mg/ha) are found in the Pacific Northwest, California, Rocky Mountains, upper peninsula of MI, and New England. The highest C stock densities for SOC (> 80 Mg/ha) are found in the upper Lake States, Pacific Northwest, northern New England, and coastal areas of the Southeast. In order to better appraise areas subject to varying C stock dynamics, each pixel was assigned to one of three categories, indicating where it had the largest proportion of its total C stocks apportioned: 1) live biomass (live tree and understory AG and BG), 2) SOC, and 3) dead wood and forest floor (Figure 9). Live biomass is the dominant C stock along the West Coast and Appalachian Mountains. In contrast, SOC is the dominant C stock along Southeastern coastal areas, New England, Great Lakes, and Great Plain’s forests. The dead wood and forest floor is dominant in areas of the Rocky Mountains.

**Figure 10:**
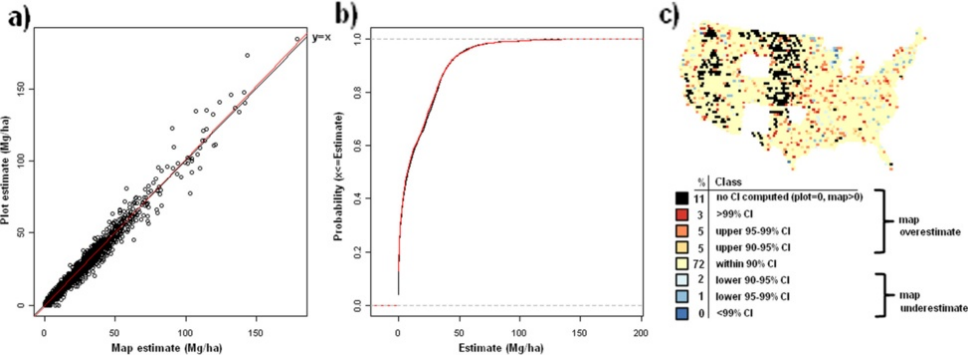
**Scatterplot, (a), cumulative distribution, (b), and map of confidence intervals of differences (c), between map-based and plot-based estimates of live tree aboveground carbon.** Results are based on C density at the spatial scale of 50 km (216,500 ha hexagons).

**Figure 11:**
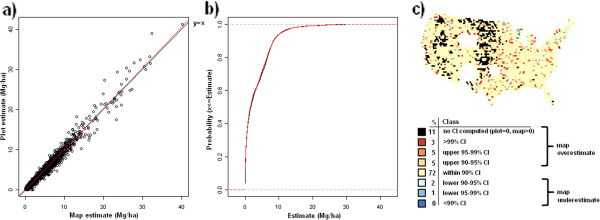
**Scatterplot, (a), cumulative distribution, (b), and map of confidence intervals of differences (c), between map-based and plot-based estimates of live tree belowground carbon.** Results are based on C density at the spatial scale of 50 km (216,500 ha hexagons).

**Figure 12:**
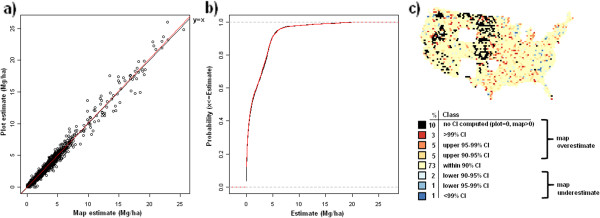
**Scatterplot, (a), cumulative distribution, (b), and map of confidence intervals of differences (c), between map-based and plot-based estimates of downed dead tree carbon.** Results are based on C density at the spatial scale of 50 km (216,500 ha hexagons).

**Figure 13:**
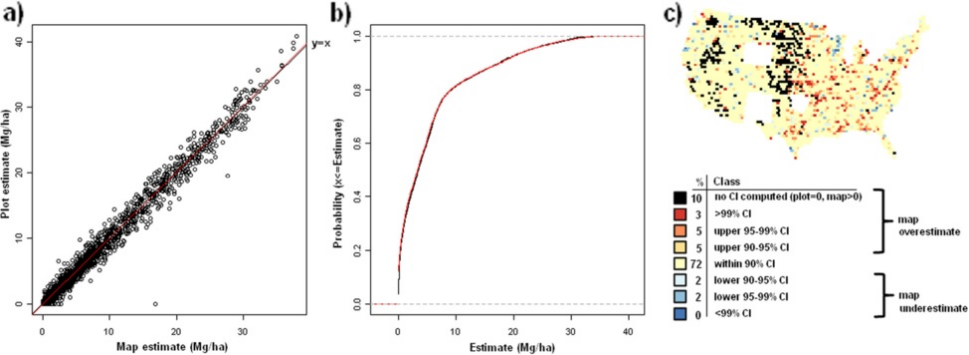
**Scatterplot, (a), cumulative distribution, (b), and map of confidence intervals of differences (c), between map-based and plot-based estimates of forest floor carbon.** Results are based on C density at the spatial scale of 50 km (216,500 ha hexagons).

**Figure 14:**
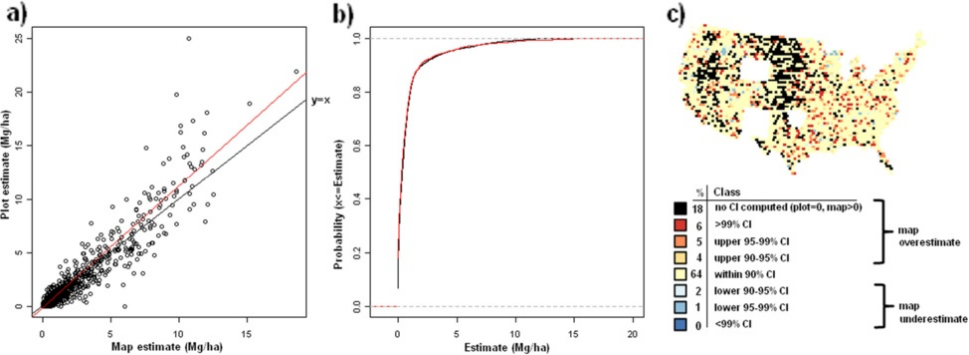
**Scatterplot, (a), cumulative distribution, (b), and map of confidence intervals of differences (c), between map-based and plot-based estimates of standing dead tree aboveground carbon.** Results are based on C density at the spatial scale of 50 km (216,500 ha hexagons).

**Figure 15:**
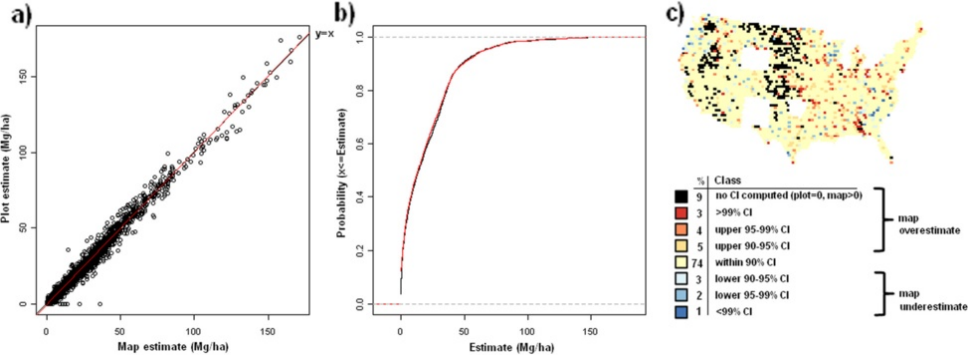
**Scatterplot, (a), cumulative distribution, (b), and map of confidence intervals of differences (c), between map-based and plot-based estimates of soil organic carbon.** Results are based on C density at the spatial scale of 50 km (216,500 ha hexagons).

**Figure 16:**
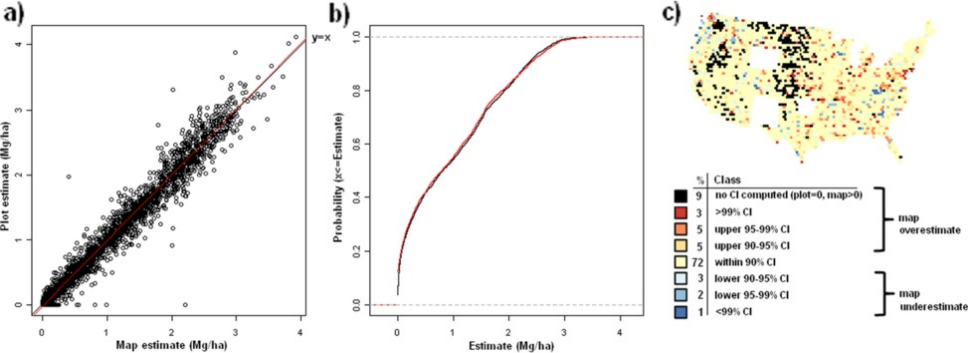
**Scatterplot, (a), cumulative distribution, (b), and map of confidence intervals of differences (c), between map-based and plot-based estimates of understory aboveground and belowground carbon.** Results are based on C density at the spatial scale of 50 km (216,500 ha hexagons).

**Figure 17:**
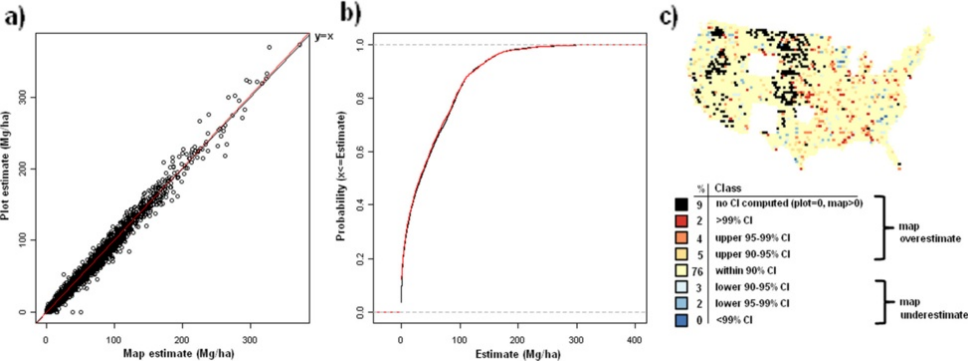
**Scatterplot, (a), cumulative distribution, (b), and map of confidence intervals of differences (c), between map-based and plot-based estimates of total forest carbon.** Results are based on C density at the spatial scale of 50 km (216,500 ha hexagons).

Validation metrics suggest good agreement between map-based and field plot-based estimates of C density across pools and spatial scales (Table 1). The strongest agreement according to all three validation metrics is at the coarsest spatial scale (200 km) with slight reductions in agreement statistics down to the finer spatial scale (50 km). At 25 km, most pools demonstrate a more substantial drop in agreement, albeit most statistics still indicate strong agreement (e.g., agreement coefficient above 0.90). The one exception is the standing dead tree C pool which had good agreement at the spatial scale of 100-200 km, but demonstrated a marked decline in agreement down to the finest spatial scale of 25 km (e.g., agreement coefficient = 0.67). The distribution curves and CI maps (Figures 10, 11, 12, 13, 14, 15, 16 and 17) reinforce the fit statistics: there is fairly robust agreement between the map-based and field plot-based estimates of C density by pool at the finer spatial scale of 50 km (216,500 ha). The differences appear to be distributed in a rather spatially unbiased manner across the conterminous U.S., although there is a tendency for the model to overestimate forest C at the edges of the forested extent. The same phenomenon was present and discussed in the earlier study of species relative abundance [13]. This is most likely an effect of the spatial mismatch between pixels and plots, as well as a mismatch between the “forest” stratum used during imputation and the FIA definition of forest land, that includes a component based on land use not readily detected using remote sensing data.

**Table 1:** Validation metrics by scale and forest C stock

**Metric**	**Scale**	**Total**	**Above-ground**	**Below-ground**	**Understory**	**Standing dead**	**Down dead**	**Forest Floor**	**Soil organic**
Agreement Coefficient^a^	200 km	0.9911	0.9942	0.9944	0.9909	0.9399	0.9932	0.9947	0.9828
	100 km	0.9943	0.9926	0.9925	0.9866	0.9379	0.9924	0.9943	0.9933
	50 km	0.9866	0.9785	0.9785	0.9697	0.8635	0.9844	0.9850	0.9850
	25 km	0.9610	0.9295	0.9300	0.9220	0.6707	0.9534	0.9605	0.9639
KS Statistic^b^	200 km	0.0364	0.0424	0.0485	0.0364	0.0485	0.0364	0.0364	0.0424
	100 km	0.0274	0.0316	0.0316	0.0247	0.0521	0.0288	0.0233	0.0288
	50 km	0.0866	0.0882	0.0882	0.0863	0.1135	0.0866	0.0863	0.0863
	25 km	0.1246	0.1260	0.1260	0.1244	0.1591	0.1244	0.1244	0.1244
RMA Slope^c^	200 km	1.0073	1.0039	1.0050	0.9941	1.1515	1.0122	1.0003	1.0089
	100 km	1.0012	1.0025	1.0027	0.9995	1.0466	1.0050	1.0059	0.9987
	50 km	1.0102	1.0171	1.0174	1.0140	1.1405	1.0151	1.0114	1.0073
	25 km	1.0248	1.0405	1.0411	1.0403	1.2158	1.0319	1.0271	1.0206

^a^ Agreement Coefficient (larger values indicate better agreement, min = 0, max = 1).

^b^ Kolmogorov–Smirnov Statistic (smaller values indicate better agreement, min = 0, max = unbounded).

^c^ Reduced Major Axis Slope (values closer to 1 indicate better agreement, min, max = unbounded).

## Discussion

This study demonstrated that a spatially explicit imputation approach may be applied to a standard forest inventory to efficiently produce continuous maps of forest C stock estimates in a timely manner. Across spatial scales ranging from 25 to 200 km, imputed C stock estimates closely matched those derived from the forest inventory data that serve as the basis for the U.S.’s NGHGI. Thus, the opportunity exists to down-scale a NGHGI to finer scales (e.g., sub-county scale). As various studies [5,10] have recently highlighted the role that forest management may play in mitigating climate change, the accurate carbon assessment of “project-scale” forest management activities is paramount to forecasting potential mitigation benefits, if any. A project scale C inventory that is consistent with regional and NGHGI’s may allow for robust verification, a desirable attribute of forest C projects [15]. In addition, consistency in monitoring forest C across spatial scales may be integral to fully understanding the dynamics of forest C from stands to ecosystems [16]. Beyond the spatial scale, such imputed maps may be rapidly updated annually to incorporate recent disturbance information. It is expected that large scale disturbance events may increasingly affect forest C stocks in the face of climate change [17]. Disturbance events may occur on annual time steps such that forests become net emitters of C [18] over a relatively short period of time. Recent wildfires and insect outbreaks [19] in western North America have highlighted the need to provide temporally continuous monitoring of forest C stocks. Whether the PGNN approach or alternative is used, spatially explicit imputation approaches enable the use of annual forest inventories to inform real-time and real-world C management situations.

Although an imputed forest C map may provide a reasonable down-scaling of a NGHGI, the situation remains that most NGHGI’s have high variability at fine scales such that the statistical power to detect stand-level change is limited [20]. Therefore, while an imputed C estimate for an individual pixel may be consistent with a NGHGI, the level of uncertainty associated with that one pixel will be very high. It may not matter how consistent a map is with a NGHGI, if the NGHGI itself contains tremendous uncertainty. Another limitation to imputing NGHGI data to maps is that dedicated analytical staff is needed to produce these outputs on an annual time step. Indeed, if the value in such an approach is its sensitivity to recent disturbance events, then likewise forest analysts will need to develop and apply imputation models within the same time frame. Finally, an important component of the PGNN approach used in this study is that the live tree attribute of basal area was a central dependent variable [13]. The imputed maps of standing dead tree C density had the poorest agreement with the empirical inventory present in the NGHGI. It has already been demonstrated in the U.S.’s NGHGI that detrital C models based on live tree attributes may substantially over/under-estimate actual C stocks at finer scales (e.g., plot-level) [12]. This may likewise occur with spatial imputation models and it is suggested that future research explore alternative imputation models for the non-living C stocks in forest ecosystems. There was reasonable agreement between the imputed estimates of SOC and the forest floor C densities compared to the NGHGI because the NGHGI currently uses models based partially on live tree attributes to estimate these stocks [6]. However, a comparison between the first empirical inventory of forest floor C stocks across the U.S. [21] and the map in this study (Figure 7) highlights the reduced uncertainty that could be realized by both adopting empirical measurements of C pools within a NGHGI and adapting imputation models to fit those unique ecosystem components.

Can imputed forest C maps inform our knowledge regarding the dynamics and attributes of C across the U.S.? Perhaps the dynamics of forest C is best illustrated by Figure 9. It is obvious that the predominant forest C pool varies by ecosystem across the U.S. While at high latitudes or in coastal/wetland areas it may be the SOC and forest floor pools that require the most attention when it comes to management and monitoring, it can be the AG biomass (whether dead or alive) that should be a focal point in most other areas. Most telling was the dominance of detrital forest C pools in most areas of the Intermountain West. The value of imputed forest C maps may be beyond monitoring C monitoring efforts at scales ranging from national to sub-county, rather it may be in identifying emerging research areas and ecological “hotspots” [21] such as areas where forest C stocks may be responding to climate change events or how detrital and soil organic C stocks may be related.

## Conclusions

Down-scaling forest NGHGI’s to finer scales (i.e. project level) is needed to provide project verification that is regionally consistent while at the same time refining the science of forest C monitoring. A map-based imputation approach, such as the PGNN technique applied in this study, affords an efficient and timely method for producing spatially continuous maps of diverse forest C pools that are consistent with a NGHGI. The uncertainty associated with each imputed pixel is dependent on not only the imputation model, but also on the models that underlie the NGHGI. We suggest that further research in both modeling areas be undertaken to refine forest C maps in the future. Until such refinements occur, the maps produced in this study can only provide rough guidance for forest C projects while highlighting the regional differences in various C pools and their associated dynamics that in turn may guide future research.

## Methods

### Data

The FIA program is the primary source for information about the extent, condition, status, and trends of forest resources in the United States [22]. FIA applies a nationally consistent sampling protocol using a systematic design covering all ownerships across the U.S., at a base national sample intensity of one plot per 2,428 ha. Land area is stratified using aerial photography or classified satellite imagery to increase the precision of estimates. Remotely sensed data may also be used to determine if plot locations are forested and should be measured in the field. FIA defines forested land as areas that have at least 10 percent tree canopy cover, are at least 0.4 ha in size, and are at least 36.6 m wide [23]. FIA inventory plots consist of four, 7.32-m fixed-radius subplots spaced 36.6 m apart in a triangular arrangement with one subplot in the center [8,24]. All trees (live and standing dead) with a diameter at breast height (dbh) of at least 12.7 cm, are inventoried on forested subplots. Within each subplot, a 2.07 m microplot offset 3.66 m from subplot center is established where only live trees with a dbh between 2.5 and 12.7 cm are inventoried.

The field data for this study were taken entirely from the FIA database [8] using the most recent annual collection (what FIA refers to as an “evaluation”) of forest inventory plots available at the time of the study for the conterminous 48 states (N.B., annual plots from western Oklahoma, New Mexico, and Wyoming were not available at the time of this study). The data collection period for the annual inventories conducted in most of the states used in this study was initiated since 2000 and ran through 2009, with a full cycle of plots being collected over a 5-year period in the East (roughly 2005-2009) and a 10-year period in the West (roughly 2000-2009). The collection of annual plots used for each state in this study contained all plots associated with that “evaluation” from the FIA database. This includes non-forested plots and, by definition, only the data collected from the most recent observation of each plot. Sample intensities vary by state, since some have not yet completed data collection for a full cycle of plots, while others have chosen to intensify their sampling intensity by 2-3 times the base intensity. FIA field data, with approximate plot locations, are freely available for download from the program’s website [25].

These field data were used in conjunction with data extracted at each plot location from a 250 m pixel resolution raster stack. This predictor dataset included vegetation phenology information derived from a time series of vegetation indices based on MODIS satellite imagery (2002-2008), mean monthly climate characteristics from the Daymet climatological model of interpolated climate station observations (1980-1997) [26], topographic metrics from the Elevation Derivatives for National Applications digital elevation model, and Omernik’s Level III ecoregions (or ecological zones) [27]. For a more complete description of these datasets and how they were used in the study, see Wilson et al. [13].

### Plot-level carbon estimates

Plot-level estimates of forest C stocks are a combination of empirically measured tree/site attributes combined with a series of individual tree/site models. Both standing live and dead tree AG C stocks are estimated using the Component Ratio Method (CRM) [9]. Briefly, the CRM facilitates calculation of tree component biomass (e.g., tops and limbs) as a proportion of the bole biomass (determined through field measured species, diameter, and often height measurements) based on component proportions from Jenkins et al. [28]. Structural deductions (e.g., loss of limbs) and wood density reductions [29] are applied to standing dead trees to account for their inherent loss of wood density and tree components through decay processes [12]. The live BG C stocks are a modeled function of the AG live tree C stocks. Understory (both AG and BG) are modeled as a proportion of the live tree AG and BG stock. The remaining forest C stocks (i.e. forest floor, downed dead wood, and SOC) are modeled as a function of a plot’s forest type, stand age, and ecoregion . While a series of empirical measurements (e.g., tree diameter and species composition) are independent variables employed by a series of models (i.e., live tree volume models to soil organic carbon models) to determine plot-level C stocks by pool, these resulting estimates are used as the empirical basis for imputation in this study. As the measurement/model details vary by individual pool, the NGHGI documentation should be referenced for specific model variables and coefficients [6].

### Phenological gradient nearest neighbor technique

To briefly summarize the methodology described in Wilson et al. [13], CCA models were developed, based on a 1/8th subsample of the plots, that related the multivariate response variable measured on the field plots (live tree basal area by species) with the associated 21 predictor variables extracted from the raster stack at each plot location. For this study, two separate CCA models were constructed: one each for the eastern and western conterminous United States, using only the plots and pixels found within the respective states and with the central states from North Dakota to Texas being included in each model. The resultant predictor variable loadings (coefficients) were used to transform the predictor variables into a featurespace of “canonical variates” that maximized the inertia in the response data that could be explained by the predictor data. This featurespace was then used to measure proximity between pixels and plots (more precisely, the pixels containing plots), and thereby to assign a plot label to each pixel in the study area. In an effort to predict a value for each pixel while minimizing the root mean squared prediction error, a small number of *k* nearest neighboring plots were used to impute a weighted mean value to each label plot, with the weight assigned to each neighboring plot based on its proximity to the label plot as measured in the featurespace of canonical variates. As in [13], a value of *k* = 7 and an inverse distance weighting exponent of 1.75 were used to produce the maps in this study. Furthermore, to account for the mismatch in spatial resolution between plots and pixels, a finer spatial resolution dataset of estimated tree canopy (i.e., 30 m pixel resolution National Land Cover Database tree canopy cover) was used to stratify the plots into “forest” (> = 25% tree canopy cover) and “non-forest” (< 25% tree canopy cover) strata during the imputation step. Finally, the predicted value for each pixel was calculated as the weighted mean of the imputed values assigned to the corresponding label plot, based independently on “forest” and “non-forest” plots, and the relative proportion of each of these strata present within the pixel.

For the purposes of the current study, all of the model outputs from the earlier study [13] were used: the plot identification (label) raster map, list of neighboring “forest” and “non-forest” plots, plot weights, and strata weights by pixel. These components were used in an analogous fashion to compute estimates of forest C stocks for each pixel using the associated plot-level measured or modeled values, restricting the *k* plots used for each estimate to the 2nd through 8th nearest neighboring plots.

### Map validation

Validation metrics used in this study were similar to those employed by Wilson et al. [13] and described in more detail by Riemann et al. [30]. First, map-based and field plot-based estimates were compared at 4 spatial scales (for convenience, these are indicated as 25, 50, 100, and 200 km) based on their spatial resolution, which is the distance between the centroids of a spatially continuous mesh of hexagons, using three validation metrics: agreement coefficient (AC), Kolmorogov-Smirnov statistic (KS), and the slope of the reduced major axis (RMA) regression line. The AC statistic [31] is symmetric and standardized describing the agreement in both datasets about a y = x line. A value of “1” indicates perfect agreement. The KS statistic quantifies the agreement between the distributions of the two datasets in terms of maximum distance between their empirical distribution functions. The KS statistic makes no assumptions about the distribution of the data and is independent of scale changes. The RMA regression line is calculated in a similar way to the ordinary least squares regression line, but with the assumption that there is error in both the x and y axes and is thus symmetrical regardless of ordering of axes. Finally, scatterplots and cumulative distribution functions of map-based versus field plot-based estimates of C density were determined for each C pool at the spatial scale of 50 km (216,500 ha hexagons). For each C pool, a corresponding choropleth map was constructed that depicts where the map-based estimate for each hexagon falls relative to plot-based confidence intervals (CI). These hexagonal choropleth maps indicate which CI (i.e., 90%, 95%, 99%, or greater) the map-based estimate falls within and whether or not the map-based estimate is an overestimate (in the upper half of the CI) or an underestimate (in lower half of the CI) relative to the plot-based estimate. Map-based estimates falling within narrower confidence intervals (e.g. 90% CI) suggest better agreement with the associated plot-based estimate than those falling within wider confidence intervals (e.g. greater than 99% CI).

## Competing interests

The authors declare that they have no competing interests.

## Authors’ contributions

BW developed the mapping methodology, constructed the CCA models, conducted the accuracy assessment, and co-wrote the manuscript. CW compiled the plot-level forest carbon density data, provided the background and analysis of the results of the study, and co-wrote the manuscript. DG conducted the imputation of plot-level data to pixels and produced the maps of forest carbon pools for the study. All authors read and approved the final manuscript.
